# INCIDENT DIFFICULTY IN INSTRUMENTAL ACTIVITIES OF DAILY LIVING: WHICH COMES FIRST?

**DOI:** 10.1093/geroni/igz038.1924

**Published:** 2019-11-08

**Authors:** Danielle L Feger, George W Rebok, Sherry Willis, Alden L Gross

**Affiliations:** 1 Johns Hopkins Bloomberg School of Public Health, Baltimore, Maryland, United States; 2 University of Washington, Seattle, Washington, United States

## Abstract

Background: Instrumental activities of daily living (IADLs) are necessary for successful independent living. Older adults may develop difficulty completing IADLs as they become physically and/or cognitively frail. The relative ordering in which IADLs deteriorate, and the importance of this ordering, is not well understood. Methods: Participants from the Advanced Cognitive Training for Independent and Vital Elderly (ACTIVE) study who reported no difficulty with IADLs at baseline were included. Individuals were followed up to 10 years for incidence of self-reported difficulty in 19 specific IADLs. The outcome of interest was time to any incident difficulty. We used Cox proportional hazards regression to estimate the hazard ratio (HR) of incident IADL difficulty for each IADL. Results: Of N=1,273 participants who contributed 6,144 person-years to the analysis, 887 developed difficulty with at least 1 IADL during the study period. The tasks in which participants reported difficulty earliest included giving self-injections (HR=5.69, [4.77, 6.79]), balancing checkbooks (HR=5.56, [4.32-7.16]), remembering often called numbers without having to look them up (HR=5.47, [4.55-6.59]), and household chores (HR=4.18, [3.43-5.11]). The last tasks to become difficult included keeping household expenses balanced (HR=0.07, [0.04-0.14]) and hanging up at the end of a phone call (HR=0.23, [0.09-0.56]). Conclusion: Independent older adults reported earlier difficulty with balancing checkbooks, remembering often called phone numbers, and doing household cleaning. Recognizing these early difficult tasks may facilitate early planning for family members and adoption of compensatory strategies.

